# Microstructure Analysis of Drawing Effect and Mechanical Properties of Polyacrylonitrile Precursor Fiber According to Molecular Weight

**DOI:** 10.3390/polym14132625

**Published:** 2022-06-28

**Authors:** Hyunchul Ahn, Hyeon Jung Gwak, Yong Min Kim, Woong-Ryeol Yu, Won Jun Lee, Sang Young Yeo

**Affiliations:** 1 Advanced Textile R&D Department, Korea Institute of Industrial Technology, Ansan 15588, Korea; hahn@kitech.re.kr (H.A.); guswnd320@kitech.re.kr (H.J.G.); 2 Department of Fiber System Engineering, Dankook University, Yongin 16890, Korea; wjlee@dankook.ac.kr; 3 Department of Materials Science and Engineering (MSE) and Research Institute of Advanced Materials (RIAM), Seoul National University, Seoul 08826, Korea; kymins@snu.ac.kr (Y.M.K.); woongryu@snu.ac.kr (W.-R.Y.)

**Keywords:** polyacrylonitrile, precursor, molecular weight, drawing, microstructure

## Abstract

Polyacrylonitrile (PAN) fiber is the most widely used carbon fiber precursor, and methyl acrylate (MA) copolymer is widely used for research and commercial purposes. The properties of P (AN-MA) fibers improve increasingly as the molecular weight increases, but high-molecular-weight materials have some limitations with respect to the manufacturing process. In this study, P (AN-MA) precursor fibers of different molecular weights were prepared and analyzed to identify an efficient carbon fiber precursor manufacturing process. The effects of the molecular weight of P (AN-MA) on its crystallinity and void structure were examined, and precursor fiber content and process optimizations with respect to molecular weight were conducted. The mechanical properties of high-molecular-weight P (AN-MA) were good, but the internal structure of the high-molecular-weight material was not the best because of differences in molecular entanglement and mobility. The structural advantages of a relatively low molecular weight were confirmed. The findings of this study can help in the manufacturing of precursor fibers and carbon fibers with improved properties.

## 1. Introduction

Polyacrylonitrile (PAN) fiber has been the most widely used precursor in carbon fiber manufacturing since the 1970s [1]. PAN fiber can be mass-produced because of its high carbon yield and stable structure. Its physical properties, efficiency, and manufacture have been widely studied [2,3]. PAN homopolymer alone is not well manufactured into fiber due to limitations concerning spinnability and thermal stability, and therefore, research on various copolymers has been conducted [4,5,6]. Methyl acrylate (MA) is the material most often used to improve the spinnability and processability of PAN materials and is considered an essential copolymer material [7,8]. In addition, various copolymers, including acids, are used for stability in oxidation and carbonization processes, and MA and itaconic acid (IA) are used in the manufacturing of commercial carbon fibers [9]. Such copolymers are indispensable in the production of carbon fibers but do affect their structural properties, and their effects on various additives can be seen in the precursor fiber stage [10]. Materials, such as lignin and cellulose have been introduced in the interest of eco-friendliness and cost reduction, and various studies have been conducted on the effect of these additives [11,12]. Although PAN-based carbon fibers have been studied in various ways for many years, the differences between their mechanical properties and those of other materials continue to motivate research on those properties, including research on molecular weight or composition, which significantly influences the mechanical properties of carbon fibers [13]. In addition, various fabrication processes related to the spinnability and mechanical properties of carbon fibers have been studied [14,15].

P (AN-MA) copolymer is typically manufactured with 3–6 wt.% MA. The processability typically improves with increasing MA, but the mechanical properties are adversely affected; therefore, an MA content higher than 6 wt.% is not recommended [10]. The limitations of carbon fiber made of P (AN-MA) copolymer pose obstacles to it being commercially mass-produced; however, it is actively used in research on the theoretical strength and mechanical properties of carbon fiber [16]. The molecular weight composition of P (AN-MA) precursor fiber has been studied extensively, as have the effects of its molecular weight on its mechanical properties [13] and changes in its properties, with changes in composition [17,18] and spinning and solidification conditions [19]. Since it is a basic component of PAN precursor fibers, it has been analyzed extensively [20], including its rheological aspects and the effects of doping treatment [21]. Since the crystallinity and structure of the fiber in the precursor fiber stage significantly affect the mechanical properties of carbon fiber, a structural study and analysis of polymer P (AN-MA) material and an understanding of the microstructural changes that occur in the fiber manufacturing process are required. There has been little research on the drawing process, especially in the PAN precursor fiber manufacturing process, which forms a structure by dispersion and diffusion of solvent in the spinning process, washing and rinsing, and the drawing process. Few studies have looked at the microstructure involved, although studies on the internal structural changes and physical property changes that occur during the stretching process have been conducted [22].

In this study, to investigate P (AN-MA) behavior under drawing conditions, the changes that occur in the mechanical behavior and properties of P (AN-MA) copolymer with changes in molecular weight were analyzed from a microstructural perspective. The PAN copolymer used for fiber spinning was polymerized to obtain various molecular weights, and the molecular weight and composition of the polymerized P (AN-MA) material were confirmed through gel permeation chromatography (GPC) and nuclear magnetic resonance (NMR) analysis to confirm the characteristics of the polymerized material. The polymerized material was spun and drawn through a wet-spinning process and a dry stretching process, respectively. Changes in the physical properties before and after stretching with changes in the molecular weight of the manufactured fibers were verified. Through image analysis, it was confirmed that there were no singularities before or after the stretching of each fiber and other factors affecting the mechanical properties. In addition, the crystallinity and void structure of each fiber was analyzed by x-ray diffraction (XRD) analysis. The effects of changes in the molecular weight of P (AN-MA) fibers manufactured by the same process on the microstructure of carbon fibers were examined, and microstructural differences in fibers with different molecular weights were investigated.

## 2. Materials and Methods

### 2.1. Materials and Polymerization

The PAN copolymer was polymerized by the typically used basic solution polymerization process [14,23]. The materials used were acrylonitrile (AN, Sigma-Aldrich, St. Louis, MO, USA) and methyl acrylate (MA, Sigma-Aldrich, St. Louis, MO, USA). Dimethyl sulfoxide (DMSO, Dajung, Siheung, Korea) was used as the solvent, and the solvent was used in the polymerization, as well as the dope preparation and spinning. Polymerization was conducted using a,a’-azobis-isobutyronitile (AIBN, Sigma-Aldrich, St. Louis, MO, USA) as an initiator. The composition of the copolymer was polymerized based on a 97:3 ratio. The polymerization was synthesized by DMSO-based solution polymerization, targeting three different molecular weights with the same composition to examine the effects of molecular weight. The molecular weight was controlled by controlling the content of the initiator AIBN, and the amount was controlled based on consideration of the polymerization environment. The polymerized material was washed and dried using distilled water and methanol and then dried at 100 °C after pulverization. The dried sample was stored in powder form until spinning and dried once more before spinning to remove as much moisture as possible.

### 2.2. Fiber Spinning and Drawing

**Figure 1:**
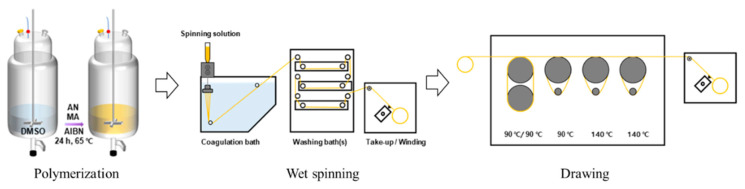
Schematic of the process of wet-spun PAN fiber.

**Figure 2:**
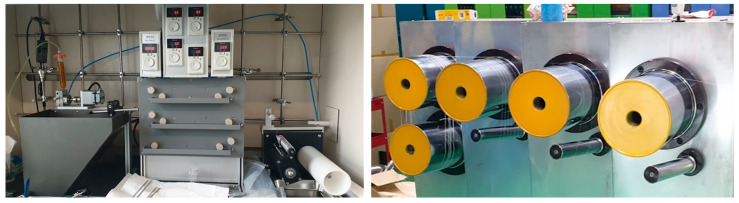
Lab-scale wet-spinning and drawing equipment.

P (AN-MA) precursor fibers were manufactured through spinning, washing, and stretching processes. A dope solution with a concentration of 25 wt% was prepared and spun, as suggested in previous fiber spinning research [8,22]. DMSO was used for both the dope solvent and the coagulation bath, and the fiber formation conditions were controlled using distilled water and DMSO at a ratio of 1:1 for the coagulation bath. After coagulation, all washing processes were performed in distilled water, and both the coagulation and washing tanks were maintained with phase silver. After washing with water, the wound fibers were immersed in distilled water for at least 24 h to remove the solvent. After drying the stored fibers, drum-type thermal stretching was performed, and stretching was performed at a temperature of 135–140 °C in stages. The hot draw ratio was six, and the draw ratio for the entire process was 18. A schematic of the polymerization, spinning, and stretching is shown in Figure 1, and the laboratory-scale equipment is shown in Figure 2.

### 2.3. Characterization

To analyze the chemical structure of the P(AN-co-MA) polymer, 400-MHz ^1^H NMR (nuclear magnetic resonance, AVANCE Ⅲ HD, Bruker, Billerica, MA, USA) analysis was performed by integrating the signal ranges of the H protons of methine (CH), methylene (CH_2_), and methyl (CH_3_) contained in the copolymer. The relative numbers of hydrogens appearing in each peak were compared, and the following formula (Equation (1)) was developed. The molar ratio was calculated according to the molecular weight of the P(AN-co-MA) polymer, measured using Waters GPC (gel permeation chromatography, Agilent 1200S, Agilent, Santa Clara, CA, USA) to determine the weighted average molecular weight (Mw) and the polymer distribution index (PDI). To confirm that the polymer was suitable for use as a spinning solution at high concentrations, P (AN-MA) polymers with different molecular weights were prepared at 25 wt%, and the viscosity was measured using a rotary rheometer (ARES G2, TA, New Castle, DE, USA) at room temperature (RT, 25 °C) and shear rates ($\overset{\cdot }{\gamma }$) of 0.01–900 1/s.

(1) $$\mathrm{Relative}\;\mathrm{mole}\;\mathrm{MA}=\alpha \cdot \left(\frac{\int CH_{3}}{3}\right)\;\mathrm{Relative}\;\mathrm{mole}\;\mathrm{AN}=\alpha \cdot \left[(\frac{\int CH_{2}}{2}\right)-\left(\frac{\int CH_{3}}{3})\right]\;\mathrm{at}{\;}^{1}H\;\mathrm{NMR}$$

The surface structure of the fibers was observed using an electron microscope (FE-SEM, SU8000, Hitachi, Tokyo, Japan), and the spinning and fiber dimensions were confirmed through the images. Chemical bonds and residual solvents in the polymers were examined by Fourier-transform infrared spectroscopy (FT-IR; Nicolet 6700; Thermo Fisher Scientific, Waltham, MA, USA). The microstructure of the fiber was measured through wide-angle X-ray diffraction (WAXD) (D8 Discover, Bruker, Billerica, MA, USA) and small-angle X-ray scattering (SAXS) (Xeuss2.0, Xenocs, Grenoble, France) with a radiation wavelength of 0.154 nm (Cu Kα). Measurement of microstructures in fibers through WAXD and SAXS has been conducted in previous studies [24,25]. In particular, the crystallinity, crystal size, and crystal orientation in fibers have been measured using two-dimensional (2D) WAXD [26,27]. In this study, as in previous studies, the microstructure in the fiber was measured using 2D WAXS. Nanoscale structures among microstructures in fibers can be measured using SAXS [27,28]. Finally, the linear density and mechanical properties of the fibers were measured using a single fiber tester (FAVIMAT, Textechno, Mönchengladbach, Germany). Measurements were performed at 20 mm/min at a 20-mm gauge length, and tensile tests were conducted in each of the 20 or more experiments.

## 3. Results and Discussion

### 3.1. Polymerization

**Figure 3:**
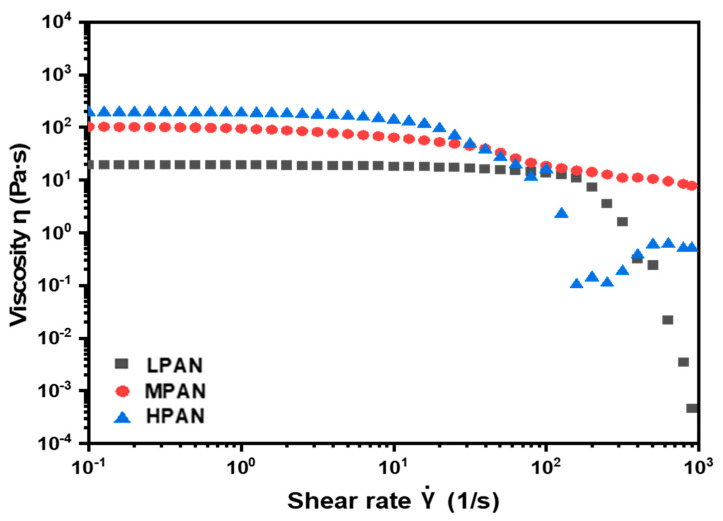
Viscous behavior of each PAN copolymer dope.

Table 1 shows the results of the ^1^H NMR and GPC analyses of the compositions and molecular weights of the P (AN-MA) polymers prepared by solution polymerization. The Mw of 150 k (LPAN) and 300 k (HPAN) were higher at 191 k and 337 k, respectively, and the Mw of 250 k (MPAN) was lower with a Mw of 221 k. However, all PDI polymers were uniformly polymerized to within 2, and the difference in molecular weight was distinct, which is believed to have affected the mechanical properties of the P (AN-MA) polymer and fiber depending on the molecular weight. To check whether wet spinning was possible when preparing a spinning solution with a high concentration of P (AN-MA) polymer, the viscosity of the spinning solution prepared at 25 wt% was measured at shear rates of 0.01–900 1/s at RT. At a shear rate of 1/s, the viscosity according to molecular weight was measured to be 19.09 Pa·s for LPAN, 94.45 Pa·s for MPAN, and 188.29 Pa·s for HPAN. It can be seen in Figure 3 that the viscosity of P (AN-MA) increases rapidly as the molecular weight increases, and although the viscosity of HPAN is high, it is a suitable viscosity for wet spinning. In addition, the viscosity increases rapidly in the high-molecular-weight sample due to the nature of the viscosity affected by the high molecular weight content.

### 3.2. Microstructures

**Figure 4:**
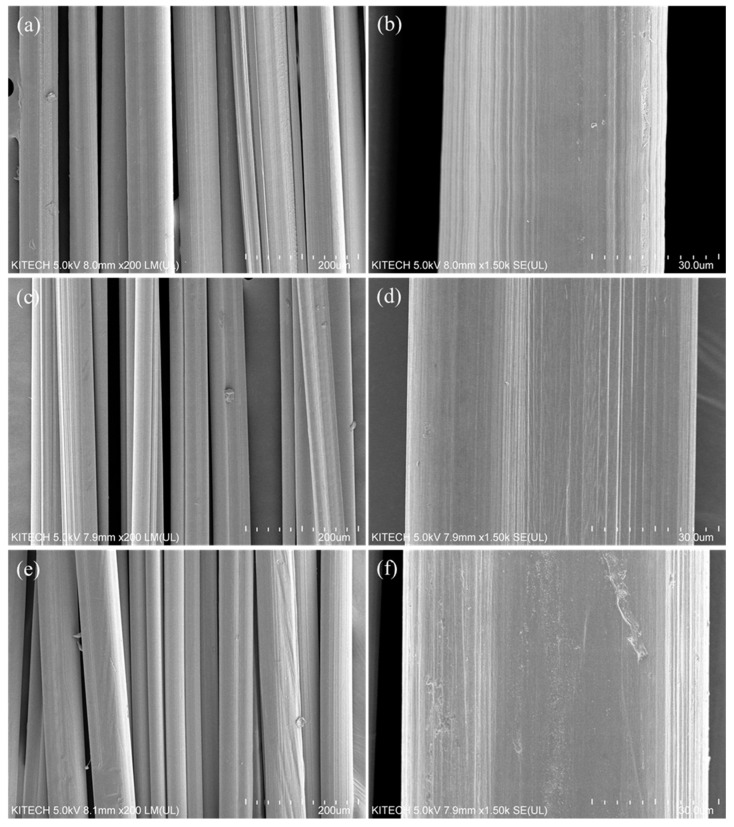
Scanning electron microscopy (SEM) images of as-spun P (AN-MA) precursor fibers; (**a**,**b**) LPAN, (**c**,**d**) MPAN, and (**e**,**f**) HPAN.

**Figure 5:**
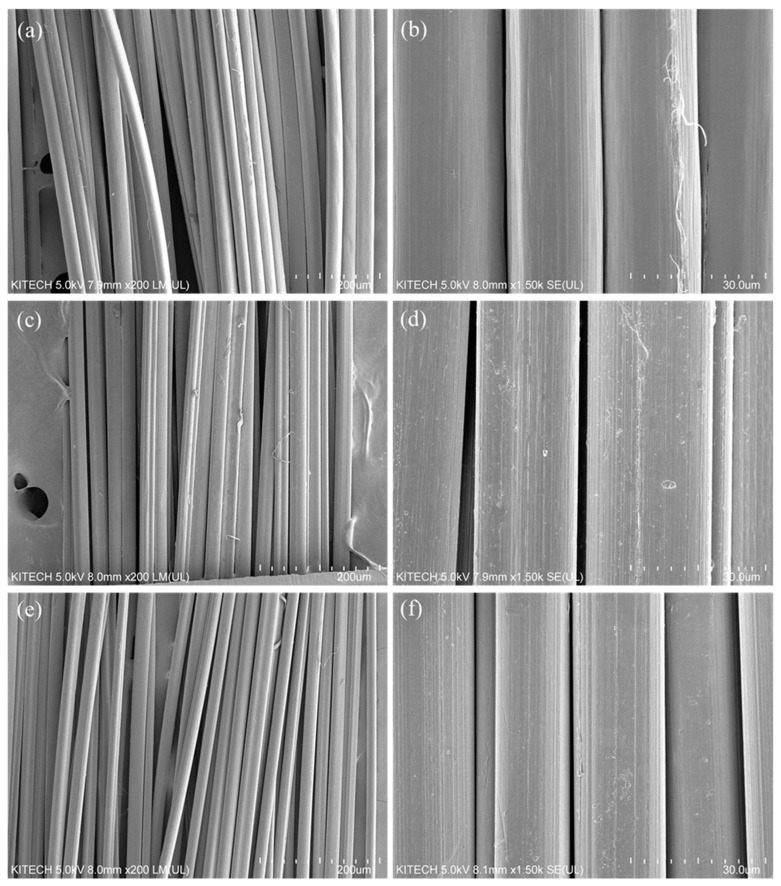
Scanning electron microscopy (SEM) images of drawn P (AN-MA) precursor fibers; (**a**,**b**) LPAN, (**c**,**d**) MPAN, and (**e**,**f**) HPAN.

To assess the effects of differences in the molecular weight of P (AN-MA) polymers on the surface shape during precursor fiber formation, the surfaces of as-spun and six-time drawn fibers of P (AN-MA) precursor fibers with different molecular weights were observed with an electron microscope (SEM). The results are shown in Figure 4 and Figure 5. On the surface of the as-spun fiber, the fibers are spun uniformly and stably, even when the molecular weight is increased. The fiber shape was manufactured in a slightly heterogeneous bean shape. This is because, as the molecular weight increases, diffusion is localized by molecular chains, forming branches when the solvent in the fiber diffuses into the nonsolvent. However, the bean shape is not critical, and it is considered to be within the limit within which the influence of mechanical properties is not significant. The P (AN-MA) precursor fiber, which was stretched six times through a dry thermal stretching machine, exhibited different stretching behavior depending on the molecular weight. The LPAN fiber decreased by 63.9% through drawing, MPAN by 56.8%, and HPAN by 65.6%. The diameter did not change significantly with the molecular weight, but the shape was different. The irregular surface shape was decreased. It can be observed that the MPAN fiber changed to a bean shape with gentle surface curvature. However, HPAN showed a reduced homogeneity in its shape. This trend is thought to affect the orientation of the polymer because of the difference in heat conduction between the inside and the outside of the fiber due to the crystals and molecular chains of the polymer in the fiber.

**Figure 6:**
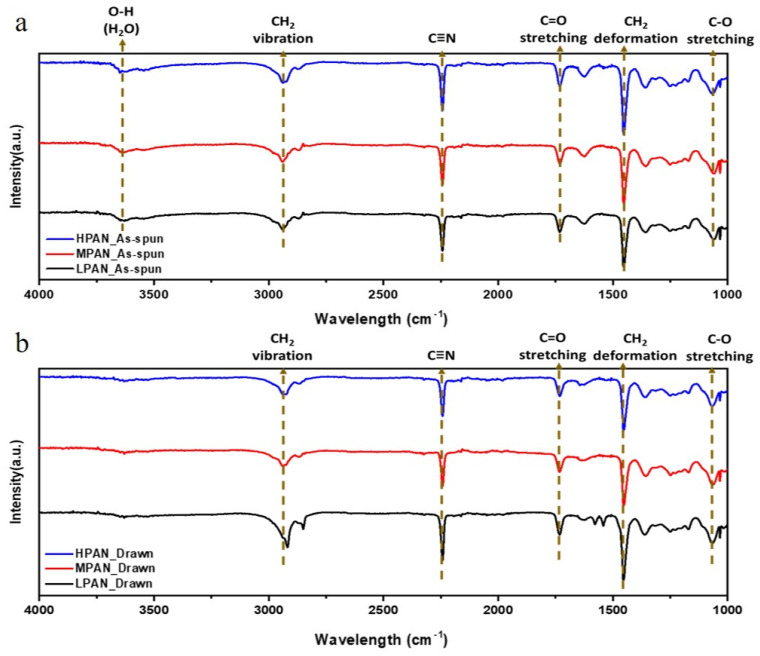
FT-IR spectra of P(AN-co-MA) precursor fibers with different molecular weights; (**a**) as-spun fibers and (**b**) drawn fibers.

Changes in the functional groups before and after the drawing of P (AN-MA) precursor fibers with different molecular weights were analyzed using an FT-IR spectrometer [29] and are shown in Figure 6. Figure 6a shows the functional group peak of the as-spun P (AN-MA) precursor fiber. The LPAN, MPAN, and HPAN of different molecular weights all show absorption wavelengths at the same position. For the main functional groups, CH_2_ vibration near 2940 and 2840 cm^−1^, C≡N (AN) near 2240 cm^−1^, C=O stretching (MA) near 1730 cm^−1^, CH_2_ deformation near 1450 cm^−1^, and C-O stretching around 1060 cm^−1^ were observed. The main difference between the drawn fiber and the undrawn fiber was that O-H (H_2_O) was observed only in the as-spun fiber at 3630 cm^−1^. The reason that this was not observed in the drawn fiber is that the moisture contained in the pores of the fiber was reduced in size, and the moisture evaporated in the process of hot drawing.

**Figure 7:**
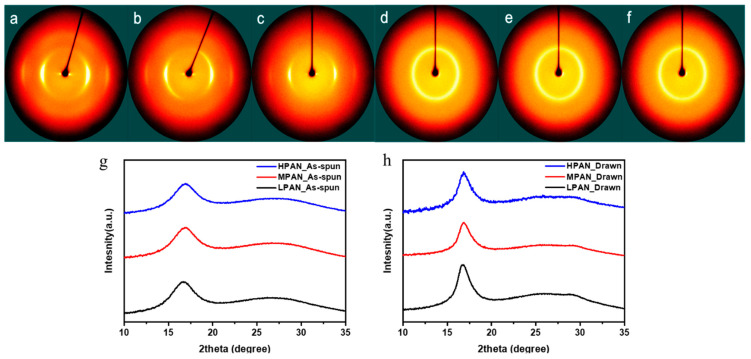
Two-dimensional wide-angle X-ray diffraction (WAXD) images of P(AN-co-MA) precursor fibers; (**a**) as-spun LPAN, (**b**) as-spun MPAN, (**c**) as-spun HPAN fiber, (**d**) drawn LPAN, (**e**) drawn MPAN, (**f**) drawn HPAN fiber, (**g**) 1D equatorially extracted XRD plot of as-spun fibers and (**h**) 1D equatorially extracted XRD plot of drawn fibers.

The microstructural changes according to the molecular weight and stretching behavior of P(AN-co-MA) precursor fibers were analyzed through WAXD and SAXS. The microstructure within the fiber can be largely divided into polymer crystal, amorphous material, and void space. Since crystallinity and amorphous regions are microscales, they are measured by WAXD. Additionally, void space is nanoscale and, therefore, is measured by SAXS. Through WAXD analysis, the crystallinity and crystal size of fibers, which are important factors that determine the mechanical properties of fibers, can be calculated. The results of the analysis are shown in Figure 7 and Table 2. The crystallinity of the fibers was calculated according to Hinrichsen’s method (Equation (2)). The crystal size of the fibers was calculated according to Equation (3). The crystallinity of the P (AN-MA) precursor fibers showed a slight difference even when the molecular weight increased, and the crystallinity increased slightly after stretching. Likewise, the crystal size was not significantly affected by the change in molecular weight, but it was confirmed that the crystal size dramatically increased through the stretching process. A strong peak appears at 2θ = 17°, which indicates (100), while a weak peak at 2θ = 27° is not evident in this result but indicates (110) [15,30]. Consequently, crystallinity can be calculated using Equation (2), where Ac is the sum of the areas of peaks and Aa is the sum of the amorphous areas [25].

(2) $$\mathrm{Crystallinity}=\frac{A_{c}}{A_{a}+A_{c}}$$

(3) $$L_{c}=\frac{K\lambda }{\beta \cos\theta }$$

Thus, crystal size can be calculated using Equation (3), where K is a constant with a value of 0.89, the wavelength (λ) used in the device is 0.154 nm, and β is the full width at half maximum (FWHM) of the strong peak [31].

**Figure 8:**
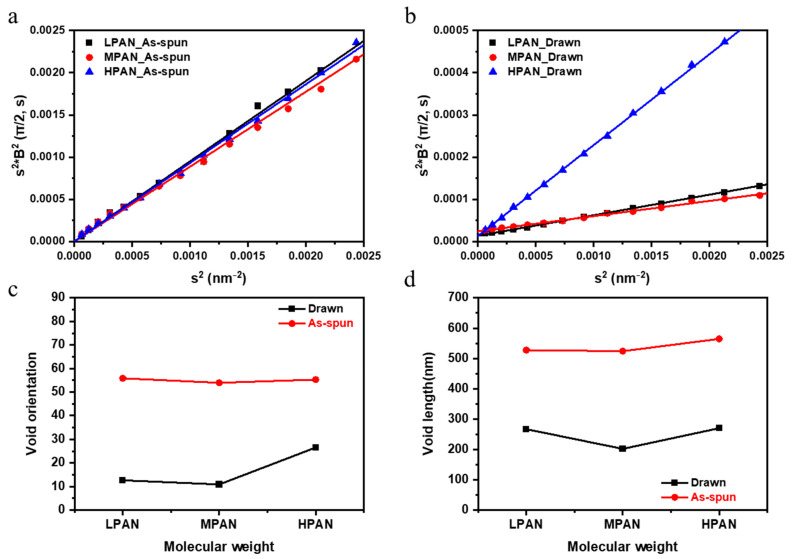
Two-dimensional small-angle X-ray scattering (SAXS); Ruland’s streak data for (**a**) as-spun fibers, (**b**) drawn fibers, (**c**) void length, and (**d**) void angle.

From a microstructural perspective, PAN fibers have nanoscale voids in the fibers that can be measured through 2D SAXS [15,25,27]. Accordingly, the void structure in the fiber is determined from the void length and void angle, depending on the process, as in the WAXD analysis. The void structure is one of the important variables that determine the properties of the fiber, appearing as a defect in the fiber, which is the starting point of breakage of the fiber. To analyze this effect, the void structure was analyzed using Ruland’s Streaks method of azimuthal scan data in various *s* (where *s* is the scattering vector) in 2D SAXS (see Figure 8). The calculation method is briefly described as follows [27]. The integral breadth Bob(s) is obtained by fitting the azimuthal distribution with the Gaussian function expressed as follows:

(4) s=|s|=2sinθ/λ

where θ is the Bragg angle, and λ is the wavelength of the incident X-ray beam (0.154 nm). The integral breadth $B_{obs}\left(s\right)$ of each azimuthal scan was calculated using Equation (5):

(5) $$B_{obs}\left(s\right)=\frac{1}{I\left(s,\;\pi /2\right)}\int I\left(s,\phi \right)d\phi$$

where Φ is the azimuthal angle, I(s, π/2) is the peak height in the azimuthal scan at *π*/2 for a particular *s*, and ∫I(s,ϕ)dϕ is the area of the peak in the azimuthal scan.

The integral breadth and the corresponding value of *s*^2^ were calculated. In addition, through the linear fitting of $s^{2}$ and $s^{2}B_{obs\;}^{2}$, the void angle was determined as the square root of the slope and the void length as the square root of the y-intercept (Equation (6)). Figure 8 shows the void structure analyzed using Ruland’s streaks method.

(6) $$s^{2}B_{obs\;}^{2}\left(s\right)=1/L_{c}^{2}+s^{2}B_{\mathrm{eq}}^{2}$$

It can be confirmed that the size of the voids increases as the molecular weight increases. In addition, as the molecular weight increases, the coagulation rate due to the non-solvent increases, and the diffusion rate of the solvent decreases due to the increase in viscosity, thereby increasing the void size. Figure 8 shows also that the shape and size of the voids can be adjusted through the stretching process. In addition, after stretching, the size of the voids decreases regardless of the molecular weight. This has to do with the stretching process. Drawing is performed at a temperature above Tg. As the chain moves, large voids disappear, and small voids become cavitation and needle-like voids. Since the mobility of the high-molecular-weight PAN chain is low, even if it is higher than Tg, the chain does not move much, so the angle is relatively less aligned.

### 3.3. Mechanical Properties

The effect of the difference in molecular weight of P (AN-MA) on the mechanical properties of precursor fibers was measured using a single-fiber tensile strength tester, and the results are shown in Table 3. The behavior of the tensile strength of the as-spun fiber was measured to be 102, 159, and 126 MPa for molecular weights of LPAN, MPAN, and HPAN, respectively. The reason that MPAN had both the highest tensile strength and greatest elongation in the as-spun is that even if the molecular weight is lower than HPAN, the spinning solution has better viscosity than HPAN. However, when the P (AN-MA) precursor fiber is drawn by 600%, the tensile strength is greatly improved as the molecular weight increases. The tensile strengths of the drawn fibers were 142, 185, and 241 MPa for molecular weights of LPAN, MPAN, and HPAN, respectively, i.e., the strength increased as the molecular weight increased. This is because the molecules in the fiber are rearranged in the fiber axis direction as the stretching progresses, and as the molecular weight increases, the interaction between the molecules becomes stronger. The elongation of the drawn fiber was almost the same for molecular weights LPAN, MPAN, and HPAN, at 11.8, 13.8, and 12.5%, respectively, and the difference in elongation behavior according to molecular weight was significantly reduced compared to the as-spun fiber. To improve the mechanical properties of the P (AN-MA) precursor fiber, it was important to increase the molecular weight of the copolymer or to manufacture homogeneous and small-diameter fibers by improving the spinning and drawing processes. In addition, it is expected that the process can be stably performed in the stabilization and carbonization process by manufacturing P (AN-MA) precursor fibers with improved mechanical properties.

## 4. Conclusions

In this study, P (AN-MA) copolymers with different molecular weights were solution-polymerized, precursor fibers were prepared through a wet-spinning process, and the properties and microstructures of the fibers were analyzed according to drawing. The molecular weights of the homogeneously polymerized P (AN-MA) copolymers were confirmed through NMR and GPC analyses. The P (AN-MA) precursor fibers exhibited differences in surface shape and properties depending on the molecular weight. In addition, the molecular structure and microstructure of the precursor fibers were analyzed according to the drawing behavior. Although molecular weight did not significantly affect crystallinity and crystal size, according to WAXD analysis, the crystal size was affected by drawing and the molecular weight. In addition, the molecular weight of the copolymer increased, as determined by SAXS analysis, and the size of the voids increased as a result of interference with the diffusion of the solvent in the fiber. As WAXD showed, the sizes and shapes of the voids could be controlled through the spinning and drawing process. In conclusion, it is possible to control the mechanical properties and microstructure of PAN-based precursor fibers by controlling the molecular weight and drawing conditions during their manufacture. The findings of this study can help in the manufacturing of precursor fibers and carbon fibers with improved properties.

## Figures and Tables

**Table 1:** ^1^H NMR and GPC analysis of P (AN-MA) copolymers.

P (AN-MA)	NMR Conversion Ratio (mol %)		GPC	
	AN	MA	Mw	PDI
LPAN	97	3	191 k	1.93
MPAN	97.7	2.3	221 k	1.86
HPAN	97	3	337 k	1.91

**Table 2:** Microstructural characterization results of PAN fibers.

Mw		Crystallinity (%)	Preferred Orientation (%)	Crystal Size (nm)
LPAN	As-spun	58.87	28.48	28.71
	Drawn	59.73	83.56	49.17
MPAN	As-spun	59.14	38.54	28.66
	Drawn	61.63	80.45	50.74
HPAN	As-spun	56.02	41.66	27.95
	Drawn	58.67	84.83	48.55

**Table 3:** Mechanical properties of PAN fibers.

Mw		Tensile Strength (MPa)	Elongation at Break (%)
LPAN	As-spun	102 ± 3	36.06 ± 4.29
	Drawn	145 ± 4	11.85 ± 1.20
MPAN	As-spun	159 ± 8	81.37 ± 6.59
	Drawn	185 ± 22	13.79 ± 0.97
HPAN	As-spun	126 ± 3	62.26 ± 9.36
	Drawn	241 ± 7	12.49 ± 1.71
